# Body attitudes and experienced early care and attachment relationships in suicidal adolescents

**DOI:** 10.1192/j.eurpsy.2022.486

**Published:** 2022-09-01

**Authors:** E. Skvortsova, L. Pechnikova, E. Zhuykova, E. Sokolova, A. Ryzhov, S. Kokorina

**Affiliations:** 1 Lomonosov MSU, Faculty Of Psychology, Moscow, Russian Federation; 2 Russian State University for the Humanities, L.s. Vygotsky Institute Of Psychology, Moscow, Russian Federation; 3 Lomonosov MSU, Departament Of Psychology, Moscow, Russian Federation; 4 G. E. Sukhareva scientific and practical center for mental health of children and adolescents of the Moscow departament of Public Health, 3d Departement, Moscow, Russian Federation

**Keywords:** adolescence, body image, attempted suicide

## Abstract

**Introduction:**

Both theoretical conceptualizations (M.&E. Laufers, E. Furman, J. Maltsberger, etc. ) and empirical studies (I. Orbach) suggest an important role body image plays in the dynamics of adolescent suicidal attempts.

**Objectives:**

To study the relationships between body image vulnerability and attachment attitudes concerning early care and current relationships.

**Methods:**

Participants were 100 adolescents with suicidal behavior (46 with suicidal ideation only, 54 with suicide attempts) compared to 100 controls (12-17 years). Body attitudes were assessed with Body Investment Scale (BIS), perceived early care was assessed by Parental Bonding Inventory (PBI), current attachments experiences were assessed with Attachment Style Questionnaire (ASQ).

**Results:**

Adolescents with suicidal manifestations scored significantly lower on BIS Body Care (p<.001) , but higher on Comfort with Touch scales (p=.05). They did not differ significantly on Body Image and Body Protection scales. With regard to perceived early care, suicidal adolescents did score lower on all Care and Control PBI scales, in both paternal and maternal forms. For current attachments suicidal adolescents scored lower on Confidence (p<.001) and higher on Approval Need (p<0.05) ASQ scales. Correlation analysis suggests, for both groups, stronger relationships of body attitude dimensions to current relationships than to perceived parental care, the former being more marked in clinical group, with Body Image scale being related to all ASQ scales ranging from r=-.32 to r=-.63.

**Conclusions:**

In current study only weaker tendency to care for body in suicidal adolescents was noted. However, in suicidal group the relationship between body image vulnerability and negative experiences of current attachments was stronger.

**Disclosure:**

No significant relationships.

